# Routine ultrasound examination by OB/GYN residents increase the accuracy of diagnosis for emergency surgery in gynecology

**DOI:** 10.1186/1749-7922-8-16

**Published:** 2013-04-30

**Authors:** Flavie Toret-Labeeuw, Cyrille Huchon, Thomas Popowski, Anne A Chantry, Alexandre Dumont, Arnaud Fauconnier

**Affiliations:** 1 Department of Gynecology & Obstetrics, Centre Hospitalier Intercommunal de Poissy – Saint-Germain, University of Versailles Saint-Quentin (UVSQ), Poissy, 78103, France; 2 EA7285, Risques cliniques et sécurité en santé des femmes et en santé périnatale, University of Versailles Saint-Quentin (UVSQ), Poissy, France; 3 INSERM, UMR S953, Paris, 75014, France; 4 Institut de recherche pour le développement (Research Institute for Development), UMR IRD 216, Paris, 75014, France

**Keywords:** Acute pelvic pain, Physical examination, Ultrasonography, Laparoscopy, Gynecologic emergency, Sensitivity, Specificity

## Abstract

**Introduction:**

Diagnostic accuracy of first-line sonographic evaluation by obstetrics/gynecology residents in determining the need for emergency surgery in women with acute pelvic pain is unknown. Aim of this study was to evaluate the diagnostic accuracy of routine ultrasound evaluation by obstetrics/gynecology residents, available 24 hours a day, in patients with acute pelvic pain.

**Methods:**

A cross-sectional retrospective study included consecutive patients who underwent emergency laparoscopy for acute pelvic pain at a teaching hospital gynecologic emergency unit, between January 1, 2004, and December 31, 2006. The laparoscopic diagnosis was the reference standard. Gynecologic and nongynecologic conditions requiring immediate surgery to avoid severe morbidity or death were defined as *surgical emergencies*. In all patients, obstetrics/gynecology residents routinely performed clinical examination and standardized ultrasonography was routinely recorded. Sonograms were re-interpreted for the study, blinded to physical examination and laparoscopic findings, according to evidence-based predetermined criteria. Sensitivity, specificity, and likelihood ratios were computed for clinical data alone, sonographic data alone, and the combination of both.

**Results:**

Emergency laparoscopy was performed in 234 patients, diagnosing 139 (59%) *surgical emergencies*. Clinical and sonographic examinations performed by the residents each independently predicted a need for emergency surgery. Combining both examinations was superior over each examination alone and had an acceptable false-negative rate of 1%.

**Conclusions:**

First-line combined clinical and sonographic examination by obstetrics/gynecology residents is effective in ruling out *surgical emergencies* in patients with acute pelvic pain.

## Introduction

Acute pelvic pain accounts for up to 40% of visits to gynecologic emergency departments (EDs) [1] and may indicate a life-threatening emergency. A prompt diagnosis is crucial to prevent severe morbidity or death [2]. The physical examination is not fully reliable [2-5]. Extensive use of diagnostic laparoscopy has been suggested to avoid missing gynecologic or non gynecologic disorders requiring emergency surgical treatment [1,6]. However, laparoscopy is an invasive procedure associated with a number of complications [7], and its use as a diagnostic tool should therefore be avoided whenever possible [8].

Since the 1990s, transvaginal ultrasonography (TVUS) has become an essential diagnostic tool for gynecologic emergencies [9]. Nonetheless, the impact of around-the-clock access to TVUS in gynecologic EDs remains unclear. In most of the studies establishing the diagnostic accuracy of TVUS in detecting gynecological emergencies, the examination was performed by board-certified radiologists or obstetricians/gynecologists. These specialized physicians are not available around-the-clock when resources are limited, as is increasingly the case in this era of patient care in the case of cost containment. It has been suggested that obstetrics/gynecology residents can perform reliable ultrasound scans in the ED to increase the rapidity and improve the quality of patient care in case of gynecologic emergencies [10].

In France, obstetrics/gynecology residents perform the initial evaluation of patients seen in gynecologic EDs, including bedside TVUS. In a previous study, we demonstrated that standardizing the gynecologic emergency ultrasonogram allowed scoring and quality control and also significantly improved the quality of ultrasonography in the gynecologic EDs [11].

The aim of this retrospective cross-sectional study was to evaluate and compare the diagnostic accuracy of first-line clinical and sonographic evaluation by obstetrics/gynecology residents available 24 hours a day in determining the need for emergency surgery in women with acute pelvic pain.

## Materials and methods

This study was approved by the CEROG (French Ethics Committee for Research in Obstetrics and Gynecology).

### Study design

We retrospectively reviewed the medical records of consecutive women who underwent laparoscopy for acute pelvic pain at the gynecologic ED of the Poissy-St Germain Hospital, France, a teaching hospital serving a large population. This historical cohort was studied between January 1, 2004, and December 31, 2006.

One resident and one senior gynecologist are available at the gynecologic ED around the clock. In France, women with acute pelvic pain are evaluated either in general EDs, in which case they are then referred to a gynecologic ED, or directly in gynecologic EDs, to which all women have free access. Thus, all patients with suspected gynecologic emergencies are seen in gynecologic EDs.

### Study population

All patients seen at our gynecologic ED for acute pelvic pain of less than 7 days’ duration and who underwent emergency laparoscopy were included. Exclusion criteria were hemodynamic shock, pregnancy of more than 13 gestational weeks, secondary laparoscopy for ectopic pregnancy initially managed with methotrexate, surgery within the last month, or virgin patients.

Among patients who did not undergo emergency laparoscopy, those who were pregnant were followed until a definitive diagnostic was made [12]. In nonpregnant patients, when the findings of all examinations were thought to be normal and the pain subsided with appropriate analgesia by the end of the visit or hospitalization, a diagnosis of idiopathic acute pelvic pain was made. After discharge, the patients were encouraged to return to our ED in case of pain recurrence.

### Study protocol

In all patients, a nurse performed an initial assessment including measurement of vital signs (Heart rate, arterial pressure and temperature), a urine hCG test and a pain intensity measurement using a Numerical Rating Scale (NRS). Then, the obstetrics/gynecology resident on duty performed standardized physical and TVUS examinations. If needed, additional investigations were performed (laboratory tests, complete ultrasound examination by a certified obstetrician/gynecologist, computed tomography). Residents were between their third and eight semester of formation in gynecology and obstetrics and were non titular of ultrasound diploma.

The senior gynecologist decided whether to perform emergency laparoscopy based on all the available data. Criteria for emergency laparoscopy were suspected adnexal torsion [13], ectopic pregnancy with a contraindication to medical treatment according to French recommendations [14], suspected tubo-ovarian abscess or peritonitis due to pelvic inflammatory disease [15], suspected massive hemoperitoneum and persistence of severe pain. For patients who did not undergo laparoscopy and before discharge, a routine time of observation of about 24 hours is usually performed in the department of gynecology.

### Data collection

The physical examination included palpation of the abdomen, speculum examination, and digital vaginal examination. The results were considered normal when there was no guarding, rebound, mass, or thickening on abdominal palpation ^2 5 16^ and no cervical motion tenderness, adnexal tenderness, or adnexal mass or thickening on vaginal examination [4,16]. If one of these features was present, the physical examination was considered abnormal.

**Figure 1:**
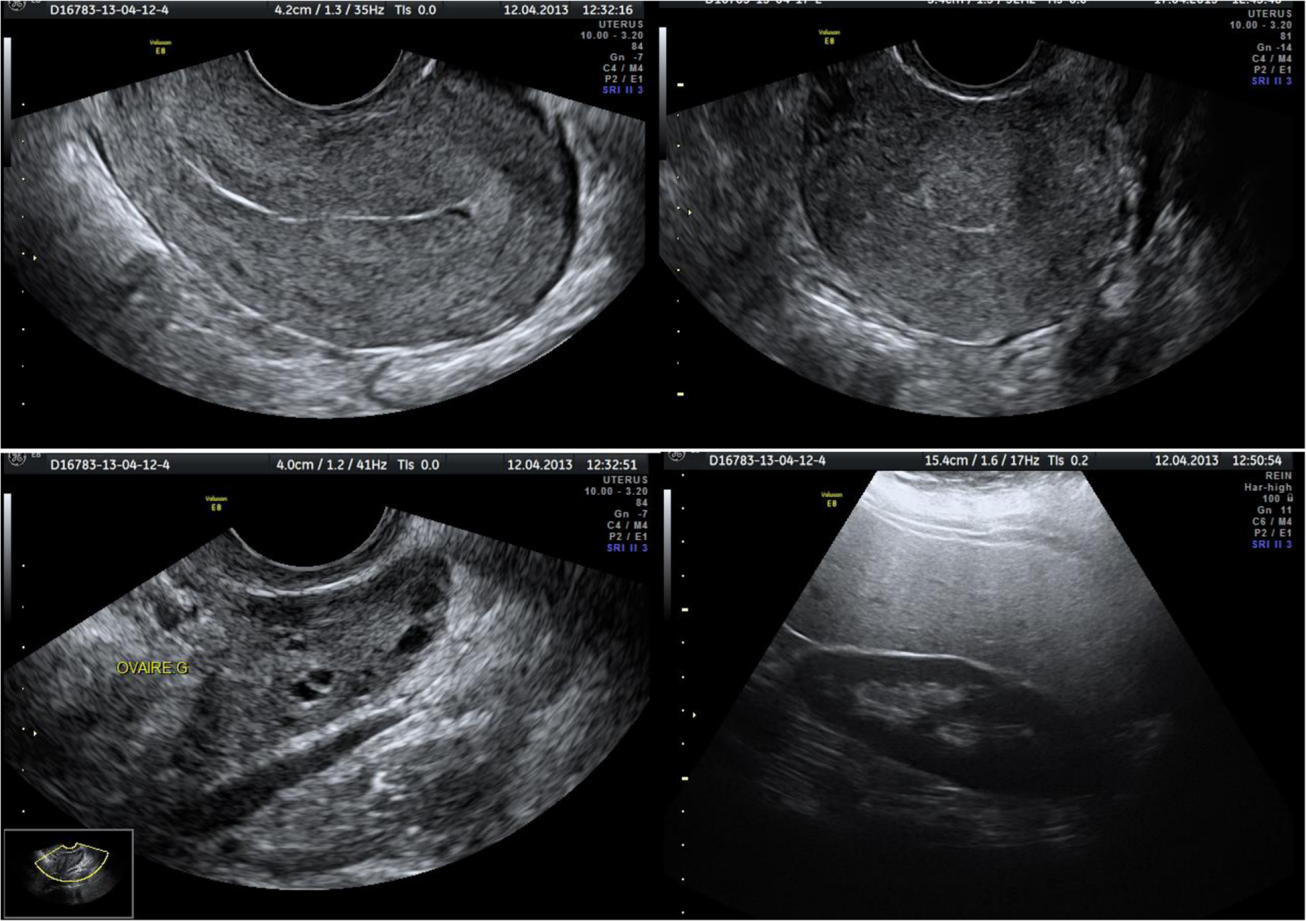
**Standardized ultrasonography scans.** (**i**) longitudinal view of the uterus, (**ii**) transverse view of the uterus, (**iii**) view of left ovary, and (**iv**) view of Morison’s pouch.

TVUS was performed using a 3.5-5 MHz transabdominal probe and a 7 MHz transvaginal probe with a General Electric Voluson 730 Expert machine (GE Medical System Europe). The residents followed a standardized TVUS protocol including at least five images, and including a routinely recording of: (i) a longitudinal view of the uterus to visualize the midline stripe indicating an empty uterus, (ii) a transverse view of the uterus, (iii and iv) a view of each ovary with the transvaginal probe, and (v) a view of Morison’s pouch with the transabdominal probe (Figure 1). One to three additional views could be obtained as dictated by the abnormal ultrasound findings (e.g., view of an ectopic gestational sac) [11]. Residents received a 1-hour class taught by a board-certified senior obstetrician/gynecologist with special expertise in gynecological ultrasonography available online (http://www.e-campus.uvsq.fr/claroline/course/index.php?cid=SAFE). This class covered image acquisition, normal and abnormal findings and image quality criteria. A copy of the written protocol for bedside emergency ultrasonography was also given to each resident.

For the present study, all sonograms were retrospectively re-interpreted by two authors: a board-certified obstetrician/gynecologist (FTL) with special expertise in gynecological ultrasonography and a research nurse (AC), who were blinded to the physical and laparoscopic findings. TVUS was considered abnormal if any of the following was seen: pelvic fluid reaching the uterine corpus or around the ovary [17], fluid in Morison’s pouch [18], abnormal adnexal mass separate from the ovary [10,19], and ovary larger than 50 mm and containing a cyst [13].

### Key outcome measures

The laparoscopy diagnosis was the reference standard. Patients were classified as having a *surgical emergency* or a *benign emergency. Surgical emergencies* were defined as gynecologic or nongynecologic disorders diagnosed by laparoscopy and associated with a high risk of complications likely to cause severe morbidity or death in the absence of appropriate emergency surgical treatment [2]. They included ectopic pregnancy with tubal rupture or active bleeding or cardiac activity or hemoperitoneum exceeding 300 mL [17]; pelvic inflammatory disease complicated by tubo-ovarian abscess or peritonitis; adnexal torsion; rupture of hemorrhagic ovarian cysts with hemoperitoneum exceeding 300 mL; appendicitis; and intestinal obstruction. *Benign emergencies*, as defined for this study, included acute conditions expected to resolve spontaneously or with appropriate medical treatment such as uncomplicated ectopic pregnancy, uncomplicated pelvic inflammatory disease, uncomplicated cyst, intra-cystic hemorrhage, myoma, endometriotic lesions, and pelvic adhesions.

### Data analysis

The preoperative physical and TVUS examinations, recorded as normal or abnormal, were compared to the laparoscopy findings as indicating a *surgical emergency* or a *benign emergency*. We used multiple logistic regression to compute the crude and adjusted diagnostic odds ratios (DORs) of having a laparoscopically confirmed surgical emergency depending on the preoperative clinical and TVUS results. The parameter values of the model were estimated using the maximum likelihood ratio method. The adjusted diagnostic odds ratios (aDORs) and their confidence intervals (CIs) were computed from the model coefficients and their standard deviations. *P* values lower than 0.05 were considered significant.

To compare the performances of physical examination alone, TVUS alone, and both in combination for diagnosing a *surgical emergency*, we computed sensitivity (Se), specificity (Sp), and the positive and negative likelihood ratios (LR+ and LR-). In the strategy including both examinations in combination, the results were considered to suggest a *surgical emergency* if the physical examination OR the TVUS OR both showed abnormalities; this strategy reflected routine use of TVUS in first line, regardless of clinical findings as we perform at our ED.

To be clinically effective and safe, a first-line diagnostic strategy had to have a low false-negative rate (i.e., sensitivity of 95% or more), with sufficient sensitivity to produce an LR- lower than 0.25. The three different strategies were compared based on the 95% confidence intervals (95% CIs) for Se and Sp according to Taylor’s formula [20]. If the point estimate of one value was not included within the 95% CI of the other, then they differed significantly with *P* smaller than 0.05. The analyses were first performed on the overall population of patients then separately in the pregnant and nonpregnant patients.

The required sample size was estimated as follows. The expected prevalence of surgical emergencies among patients who underwent laparoscopy was 50%. Using computation of the 95% CI with an unknown ratio estimator of the standard deviation, including 200 patients with laparoscopy would produce a lower limit of the 95% CI of 0.95 if the true false-negative rate is less than or equal to 2%. To take into account the occurrence of exclusion criteria and missing data in some patients, we planned to include 300 patients.

## Results

**Figure 2:**
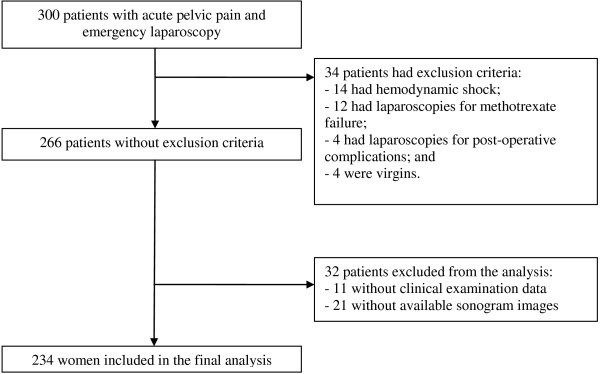
Flow chart of the study population.

Of the 300 patients who met the inclusion criteria between January 1, 2004, and December 31, 2006, 34 had one or more exclusion criteria (Figure 2). Among the 266 eligible patients, 32 had missing physical examination data or no recorded ultrasound images, leaving 234 patients for the analysis. The characteristics of the patients with missing data did not differ from those of the patients included in the analysis.

The main patient characteristics and laparoscopy diagnoses are shown in Table 1. Of the 234 patients, 139 (59%) had laparoscopically confirmed *surgical emergencies* and the remaining 95 (41%) patients had *benign emergencies* that did not require immediate surgery, including 7 (6.3%) entirely normal findings at laparoscopy.

**Table 1:** Characteristics of the study population and laparoscopy diagnoses

	**Overall population N=234**	***Surgical emergencies *N=139**	***Benign emergencies *N=95**
Age in years, mean±SD	31.3 ± 7.0	31.9 ± 6.9	30.5 ± 7.1
Gravidity, median [range]	2 [0–9]	2 [0–9]	1 [0–6]*
Parity, median [range]	1 [0–6]	1 [0–6]	0 [0–4]*
Contraception, n (%)	65 (27.9)	37 (26.8)	28 (29.5)
Pain NRS score at admission, mean±SD	6.7 ± 2.6	6.9 ± 2.6	6.4 ± 2.5
Positive hCG test, n (%)	150 (64.1)	97 (69.8)^†^	53 (55.8)^†^
**Laparoscopy diagnosis**			
Ectopic pregnancy, n (%)	136 (58.1)	91 (65.5)	45 (47.4)
Pelvic inflammatory disease, n (%)	31 (13.2)	25 (18.0)	6 (6.3)
Adnexal torsion, n (%)	15 (6.4)	15 (10.8)	NA
Appendicitis, n (%)	4 (1.7)	4 (2.9)	NA
Ruptured hemorrhagic cyst, n (%)	5 (3.0)	2 (1.4)	3 (5.3)
Other diagnosis, n (%)	36 (15.0)	2 (1.4)^‡^	34 (34.7)^‡^
Normal, n (%)	7 (2.6)	NA	7 (6.3)

*Surgical emergencies* were ectopic pregnancies with tubal rupture or active bleeding or cardiac activity or hemoperitoneum over 300 mL; pelvic inflammatory disease complicated with pyosalpinx, tubo-ovarian abscess, or pelvic peritonitis; adnexal torsion; hemorrhagic ovarian cyst rupture with hemoperitoneum exceeding 300 mL; appendicitis; and intestinal obstruction.

*Benign emergencies* were conditions expected to resolve spontaneously or with appropriate medical treatment.

NRS, numerical rating scale for pain severity; hCG, human chorionic gonadotropin; NA, not applicable; SD, standard deviation; NRS, Numerical rating scale; hCG, serum human chorionic gonadotrophin; NA, not applicable.

*P<0.05, Student’s *t* test; †*P*<0.05, Chi-square; ‡ Intestinal obstruction; ‡ uncomplicated ovarian cysts or intracystic hemorrhage.

Both the physical examination alone (DOR, 3.5; 95% CI, 1.8 to 6.9; *P*<0.001) and TVUS alone (DOR, 6.6; 95% CI, 2.8 to 15.6; *P*<0.0001) independently predicted a laparoscopy diagnosis of *surgical emergency.* However, when used alone, neither the physical examination nor TVUS performed sufficiently well to rule out a surgical emergency (Table 2). TVUS alone was better than the physical examination alone (false-negative rates, 5.8% and 13.0%, respectively). Table 3 lists the diagnoses of the false-negative results of the physical examination and TVUS.

**Table 2:** Diagnostic accuracy of physical examination, transvaginal ultrasonography, and both for diagnosing surgical emergencies

	**Physical examination alone**				**TVUS alone**				**Strategy combining physical examination andTVUS**^†^			
	Se% (n/N) [95% CI]	Sp% (n/N) [95% CI]	LR +	LR –	Se (n/N) [95% CI]	Sp (n/N) [95% CI]	LR+	LR –	Se (n/N) [95% CI]	Sp (n/N) [95% CI]	LR+	LR –
Overall population	87% (121/139) [82–93]	33% (31/95) [23–42]	1.3	0.4	94% (131/139) [90–98]	27% (26/95) [18–36]	1.3	0.2	99% (138/139) [98–100]	7% (7/95) [2–13]	1.1	0.1
Pregnant women	84% (81/97) [76–91]	42% (22/53) [28–55]	1.4	0.4	96% (93/97) [92–100]	13% (7/53) [4–22]	1.1	0.3	99% (96/97) [97–100]	6% (3/53) [0–12]	1.1	0.2
Non-pregnant women	95% (40/42) [89–100]	21% (9/42) [19–34]	1.2	0.2	91% (38/42) [82–99]	45% (19/42) [30–60]	1.6	0.2	100% (42/42) [92 – 100]	10% (4/42) [1–18]	1.1	0

Se, sensitivity; CI, confidence interval; Sp, specificity; LR, likelihood ratio.

†Corresponds to a strategy of routine TVUS regardless of the clinical findings, abnormal findings include abnormal examination OR abnormal TVUS.

TVUS, transvaginal ultrasonography; Se, sensitivity; Sp, specificity; LR+, positive likelihood ratio; LR-, negative likelihood ratio; 95%CI, 95 % confidence interval.

**Table 3:** Diagnoses in patients with a laparoscopy diagnosis of surgical emergency but had negative physical examination or negative transvaginal ultrasonography or negative with both examinations combined

	**FN, physical examination, n (%)**	**FN, TVUS, n (%)**	**FN, physical examination combined with TVUS†, n (%)**	**Total number of patients with surgical emergencies, N**
**Ectopic pregnancy**	14 (15%)	1 (1%)	0	91
**Pelvic peritonitis**	0	1 (4 %)	0	25
**Adnexal torsion**	3 (20%)	3 (20%)	1 (7%)	15
**Appendicitis**	0	1 (25%)	0	4
**Intestinal obstruction**	0	2 (100%)	0	2
**Ruptured hemorrhagic cyst**	1 (50%)	0	0	2
**Total**	18 (13%)	8 (6%)	1 (0.7%)	139

Percentages were computed by dividing the number of false negatives by the total number of surgical emergencies.

FN, False negatives; TVUS, transvaginal ultrasonography.

†Corresponds to a strategy of routine TVUS regardless of the clinical findings, abnormal findings include abnormal examination OR abnormal TVUS.

The strategy combining physical examination and TVUS in first-line was better than the strategy including only physical examination according to our criteria in which surgical emergencies were suspected based on abnormal clinical OR TVUS findings. This strategy decreased the false-negative rate from 13% (physical examination alone) to less than 1% (Table 3). The strategy combining physical examination and TVUS was the one maximizing Se and decreased negative LR to an acceptable rate of 0.1. When pregnant and nonpregnant patients were analyzed separately, the results were unchanged (Table 2).

## Discussion

According to our data, physical examination cannot be used alone to safely rule out a *surgical emergency* in a woman presenting with acute pelvic pain. Inversely when both the physical examination and TVUS are normal, the risk of a *surgical emergency* is less than 1%. This suggests the benefit of adding bedside standardized ultrasonography in the first-line diagnostic management of suspected gynecologic emergencies.

One of the strengths of our study is that TVUS findings are recorded routinely at our institution using a standardized protocol [11]. This standardized protocol, with a routine recording of standardized images, allows a reviewing of those scans, even a long time after. Recording pictures in the patient’s chart may also decrease the need for subsequent repeat ultrasonography, thereby saving time and diminishing healthcare costs. Furthermore, we did not have to rely on a written description of the TVUS findings in the medical record. The TVUS findings were determined by blinded observers using objective criteria. These criteria are reliable and have been proven useful for diagnosing specific gynecologic emergencies [9,10,13,15,21].

It has been demonstrated that the availability of TVUS at the initial assessment of both pregnant and nonpregnant women decreased patient time management, unnecessary admissions, outpatient follow-up examinations and also modified treatment decisions [22,23]. Nonetheless, we did not find any published study showing clear-cut evidence that routine ultrasonography decreases unfavorable patient outcomes. We demonstrate that including around-the-clock TVUS as a first step investigation in addition to the physical examination is an effective strategy to rule out surgical emergencies at the gynecologic ED by reducing the risk of diagnostic errors.

In France, there is at least one resident on duty around the clock with unlimited access to TVUS in gynecological EDs, even when no radiologist or board-certified obstetrician/gynecologist is available. Another particularity in France is that ultrasonography for gynecologic emergencies are under the supervision of board-certified obstetricians/gynecologists instead of radiologists. In contrast, in most of the developed countries, emergency ultrasonography is performed at the request of ED physicians by radiologists or board-certified obstetricians/gynecologists [22,23]. Although, this strategy optimizes the quality of ultrasound examination, our results suggest that suspecting surgical emergencies based on the physical examination alone does not perform well for the diagnosis of gynecologic emergencies. Instead, the French strategy of first-line ultrasonography performed by non-specialized healthcare providers should be compared with the so-called “limited” sonogram in the 2^nd^/3^rd^ trimester of pregnancy. These examinations do not replace a standard complete ultrasound examination but are performed to obtain an immediate answer to a specific clinical question [24], as FAST scanning in EDs. Bedside abdominal ultrasonography by a surgeon was also introduced several years ago as a routine examination for patients with acute abdominal pain and produced similar results, improving the rate of correct diagnoses [25].

The quality of bedside ultrasonography by obstetrics/gynecology residents is obviously not comparable to that obtained by board-certified specialists, as the quality of examination is highly variable [11]. Furthermore, experience is a key factor in the ability of transvaginal ultrasound to manage women with pelvic pain with accuracy [9]. Nonetheless, in our center, we made important efforts to implement a standardized ultrasonography protocol [11] to reduce the heterogeneity of the quality of ultrasonography performed by residents. This quality process probably increased the usefulness of bedside TVUS for the diagnosis of gynecologic emergency. One application of this process would that these scans could be performed by anyone involved in gynecologic emergencies management with appropriate training (ie ED physicians, Family Medical doctors, midwife or advanced nurse practitioners). This training should include rigorous implementation of standardized ultrasonography protocol in EDs, with quality control of ultrasonography by board-certified obstetricians/gynecologists or radiologists to obtain individual accreditation. Thus, this accreditation could decrease the heterogeneity of ultrasound examination and allow correct interpretation in order to make correct clinical decision regarding surgical emergencies.

Nonetheless, our study has several limitations. First, we were not able to have the physical examination and TVUS done by two different individuals, in contrast to another group [23]. The physical examination was performed before TVUS, and its results may therefore have influenced the recording of the images. However, calculating the conditional statistics of one examination according to the result of the other showed no differences with the main results (data not shown).

Second, our strategy of including only women who underwent laparoscopy may have led to verification bias. We chose to select patients with laparoscopy to ensure that the final diagnosis was established with certainty. However, the decision to perform laparoscopy was taken by a senior physician, based possibly on the result of the physical and TVUS findings by the resident, which may have artificially increased Se and decreased Sp of both examinations.

Third, our follow-up data on patients in whom emergency laparoscopy was deemed unnecessary may have been incomplete. We believe that the risk of missing a surgical emergency among patients who leave the ED without undergoing laparoscopy is low as pregnant women received very close follow-up after ED discharge until the hCG test became negative and patients discharged with undiagnosed surgical emergencies would eventually come back to our ED, which serves a vast geographic area.

## Conclusions

Our findings indicate that combining routine bedside TVUS with the physical examination performed by gynecology/obstetrics residents on duty around-the-clock in gynecologic EDs is more effective than physical examination alone in ruling out potentially life-threatening emergencies in women with acute pelvic pain. The use of a standardized TVUS protocol and stringent objective criteria for interpreting the images may play a role in the beneficial effects of routine TVUS.

## Consent

Written informed consent was obtained from the patient for publication of accompanying images.

## Competing interests

The authors have no conflicts of interest.

## Authors’ contributions

AF and AD design the study; Acquisition of data were performed by FTL, TP an AC, Statistical analysis were performed by TP, CH and AF; Analysis and interpretation of data were performed by AC, AD, CH and AF; FTL, CH and AF draft the manuscript; AD and AF made critical revision of the manuscript for important intellectual content; AF and CH have full access to all of the data and take responsibility for the integrity of the data and the accuracy of the data analysis. All authors read and approved the final manuscript.
